# A Novel Pathogenic Variant in NAGLU (N-Acetyl-Alpha-Glucosaminidase) gene Identified by Targeted Next-Generation Sequencing Followed by in Silico Analysis

**Published:** 2019

**Authors:** Mehdi KHORRAMI, Manijeh MAHDAVI, Fatemeh FAKHR, Majid KHEIROLLAHI

**Affiliations:** 1 Department of Genetics and Molecular Biology, School of Medicine, Isfahan University of Medical Sciences, Isfahan, Iran; 2 Pediatric Inherited Diseases Research Center, Research Institute for Primordial Prevention of Non-communicable disease, Isfahan University of Medical Sciences, Isfahan, Iran

**Keywords:** Sanfilippo syndrome type B, Mucopolysaccharidosis IIIB, Targeted exome sequencing, *NAGLU* gene

## Abstract

**Background:**

Mucopolysaccharidosis IIIB (MPS IIIB) (Sanfilippo Syndrome Type B; OMIM 252920) is an autosomal recessive metabolic disorder caused by mutations in the *NAGLU* gene which encode lysosomal enzyme N-acetyl-glucosaminidase, involved in degradation of complex polysaccharide, heparan sulfate. The disease is characterized by progressive cognitive decline and behavioral difficulties and motor function retardation.

**Materials & Methods:**

In this study, targeted exome sequencing was used in consanguineous parent (mother) of a deceased child with clinical diagnosis of mucopolysaccharidosis. Sanger sequencing was performed to confirm the candidate pathogenic variants in extended family members and segregation analysis. In silico pathogenicity assessment of detected variant using multiple computational predictive tools were performed. Computational docking using the Molegro Virtual Docker (MVD) 6.0.1 software applied to evaluate affinity binding of altered protein for its ligand, N-Acetyl-D-Glucosamine. Moreover, with I-TASSER software functional alterations between wild and mutant proteins evaluated.

**Results:**

We identiﬁed a novel heterozygote deletion variant (c.1294-1304 del CTCTTCCCCAA, p.432LeufsX25) in the *NAGLU* gene. The variant was classified as pathogenic based on the American College of Medical Genetics and Genomics guideline. Computational docking with the Molegro Virtual Docker (MVD) 6.0.1 software confirmed different affinity binding of truncated protein for its ligand. Moreover, I-TASSER software revealed structural and functional alterations of mutant proteins.

**Conclusion:**

This study expands the spectrum of *NAGLU* pathogenic variants and confirms the utility of targeted NGS sequencing in genetic diagnosis and also the utility and power of additional family information.

## Introduction

Mucopolysaccharidosis IIIB (MPS IIIB) (Sanfilippo Syndrome Type B; OMIM 252920) is an autosomal recessive metabolic disorder caused by mutations in the *NAGLU* gene, which encode lysosomal enzyme N-acetyl-glucosaminidase (1). This enzyme involved in breakdown a subset of glycosaminoglycan compound called heparan sulfate. Its impaired function leads to increased urinary excretion and accumulation of partially degraded component in tissues (2).

Over 150 different *NAGLU* mutations have been reported in the Human Gene Mutation Database (HGMD). MPS IIIB is clinically heterogeneous (3-5) but the clinical course of MPS III can be divided into three phases (6, 7). In the first phase, which usually starts between 1 and 4 yr of age, delayed cognitive development becomes apparent after an initial normal pre-natal and early post-natal of life. The second phase of the disease commonly begins around 3-5 yr and can last for 5 to 10 years. This phase is characterized by progressive behavioral difficulties ultimately leading to severe dementia. Hyperactivity, gradual intelligence decline, sleep disturbance in combination with good muscular strength make difficult to manage the affected child. In the third and final stage, the nature of illness change, behavioral problems come to end slowly and progressive motor retardation with swallowing difficulties and spasticity appear and ultimately patient regress to a vegetative state. In MPS IIIB, death usually occurs at the end of the second or the beginning of the third decade of life, but survival into the fourth decade has been reported in attenuated form (6).

In this study, using targeted exome sequencing, we identiﬁed novel heterozygote mutations in the *NAGLU* gene in consanguineous parents of a child who was deceased in her 14 years old where the diagnosis of death was mucopolysaccharidosis.

## Materials and Methods

**Subjects**

An 8-year-old girl with the clinical presentation of mucopolysaccharidosis, born to an Iranian consanguineous (first cousin) healthy parents with a healthy older sister, was referred to Medical Genetics Counseling Center of AL Zahra Hospital, Isfahan, central part of Iran. Unfortunately, the family did not follow up, and she was died in her 14 years old in 2016. 

As our subject did not undergo molecular diagnosis in her lifetime and due to her parents’ decision to have another child, and regarding that they were first cousins, we decided to study for alteration in 10 known genes in MPS in deceased child’s mother and then evaluated the detected variant or variants in other family members. Our subject’s birth history was normal and full term but birth weight was 2.5 kg. The main features observed early was hirsutism and restlessness. Since the age of about one year, she could express her meaning and walk but in the second year cognitive development decline, especially speech decline was apparent. At the age of four, she manifested motor developmental delay, behavioral problems, aggressiveness, intellectual decline, and hepatosplenomegaly. She continued to deteriorate with progression dementia, poor eye vision, brain atrophy, urinary incontinence, a decline of all motor functions, kyphosis, progressive neurologic deterioration, sleep disturbance, joint contractures, orthopedic manifestations, pelvic pain, and coarse facial features. Later, she manifested high fever, inability to swallow, low platelet and hair loss and ultimately regressed to a vegetative state until death.

This study was carried out in accordance with the International Ethical Guidelines and Declaration of Helsinki and approved by Ethics Committee of Isfahan University of Medical University. Informed written consent was taken from all family members following peripheral blood collection.

**DNA extraction and genotyping via NGS**

Genomic DNA was extracted from peripheral blood samples collected in EDTA tubes using Exgene Blood SV mini kit (GENEALL BIOTECHNOLOGY CO, LTD, South Korea) and its quality and quantity determined using agarose gel and Nanodrop 2000 instrument (Thermo Fisher Scientific Inc., USA), respectively. The genetic sequencing test was performed using a custom designed Nimbelgen chip capturing the gene panel of mucopolysaccharidosis including *ARSB, GALNS,GLB1,GNS,GUSB,HGSNAT,IDS,IDUA,SGSH,NAGLU* (Agilent Technologies, Inc., Santa Clara, CA, USA), followed by a paired-end high-throughput sequencing using Illumina HiSeq 2500 with coverage 150X mean depth (Illumine Inc., San Diego, CA, USA). All exons and flanking 10 bp were detected and analyzed. Detected variations included point mutations, deletion, and duplication (< 250). Briefly, the variant analysis was done via mapping the text files of sequence read to the reference genome (UCSC hg19) using the Burrows-Wheeler Alignment software with default parameters. Following alignment, Genome Analysis Tool Kit (GATK) software library was used to identify single nucleotide polymorphism and insertion-deletion SNPs and indels of next-generation sequencing data (8). Then, applying public databases, including 1000 Genome Project (http://www.1000genomes.org), HapMap samples, and dbSNP, variants with frequency >5% and synonymous substitutions were filtered out. Remaining variants were filtered to downstream analysis, according to their frequency, chromosomal location, mode of inheritance, functional consequences, inheritance pattern, and clinical history

**In silico pathogenicity assessment of variant and computational docking**

Bio-informatics interpretations using multiple computational (in silico) predictive tools such as MutationTaster (http://www.mutationtaster.org/) (9), SIFT Indel (http://sift-dna.org/) (10) and DDIG Indel (http://sparks-lab.org/ddig/) were performed (11). To predict the effect of detected variant on encoded protein function, N-acetyl-glucosaminidase interaction with some ligands such as N-acetyl glucose amine, beta-D-Mannose, glycerol and D-Xylitol was examined via docking stimulation using the Molegro Virtual Docker (MVD) 6.0.1 software (12). The Moldock scoring function based on piecewise linear potential and a re-ranking procedure was applied to select the best poses of the ligand. Lower energy scores indicate more favorite protein-ligand complexes. I-TASSER was used to evaluate computational structural modeling and functional alterations between wild and mutant proteins (13). The crystal structure of the N-acetyl-glucosaminidase protein (PDB 4XWH) and the modeled structure of truncated protein prepared were applied for structural analysis using homology modeling in SWISS-MODEL web server (https://swissmodel.expasy.org/) (14-17). 

**Co-segregation study**

The co-segregation of the variant with the *NAGLU *gene was done using Sanger sequencing for the family members and extended family members of the proband in order to confirm the accuracy of detected variant identified by NGS. Following primers designed via Primer 3 software (F: 5′- TCCTGGTTCTGGACCTGTT - 3′ and R: 5′-GCTCAGCCATGAGGGAATAG -3′) to amplify the region of deletion in exon 6 of all family members. The PCR products were subsequently visualized using 1% agarose gel and bidirectional sequencing was carried out by on an ABI 3130 sequencer (Applied Biosystems). The sequences were compared with the *NAGLU *gene reference sequence. Moreover, the degree of pathogenicity evaluated based on the criteria introduced by the American College of Medical Genetics and Genomics (ACMG) guideline (18).

## Results

The affected individual described in this paper was diagnosed with MPS by exact clinical evaluation but did not undergo molecular diagnosis in her lifetime. Her parents were consanguineous, therefore we studied for alteration in 10 known pathogenic genes in MPS in deceased child’s mother and then we evaluated the identified variant in proband^,^s husband and other relatives. The analytical sensitivity and specificity of NGS method used in this assay for detection of single point mutations and small Indels were assumed to be 99>%. 

Analysis of targeted exome sequencing data for proband was carried out and results revealed a novel heterozygote pathogenic variant within the coding region of the *NAGLU* gene. The variant was 11 bp deletion located in the sixth exon (c.1294-1304 del CTCTTCCCCAA, p.432LeufsX25) and caused a frameshift (Table 1). This gene encoded for as N-acetyl glucose amine, have been previously reported to cause mucopolysaccharidosis IIIB (MPS IIIB). The pathogenic effects of the c. 1294-1304 deletion on N-acetyl glucose amine, structure are presented in Table 2. Three bioinformatics programs predicted the variant will be damaging/deleterious. The Mutation Taster determined that the deletion was disease-causing through Nonsense-Mediated mRNA Decay (NMD) and probably the protein features were affected. The deleterious effect of the variant was supported by SIFT Indel (with Confidence Score of 0.858) and DDIG Indels (with a probability score of 0.617872). The length of N-acetyl glucose amine sequence was decreased from 743 amino acids to 454. 

**Figure 1:**
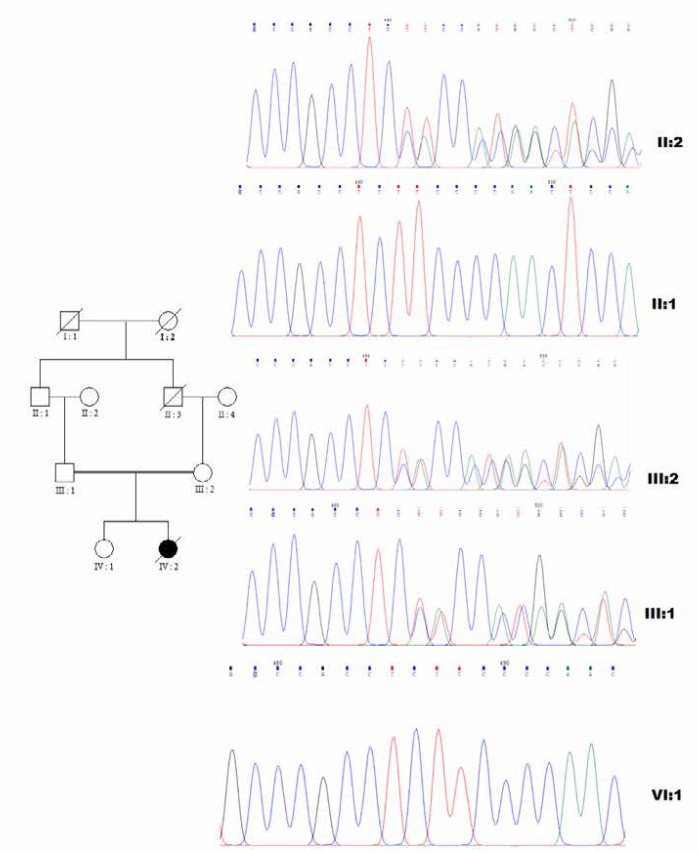
The result of co-segregation study using Sanger sequencing is consisting of autosomal recessive nature of MPS IIIB. The proband (IIІ-2), her husband (IIІ-1) and mother in law (IІ-2) are heterozygous. The proband^,^s daughter (ІV-1) and father in law (IІ-1) do not carry the mutant allele

**Figure 2:**
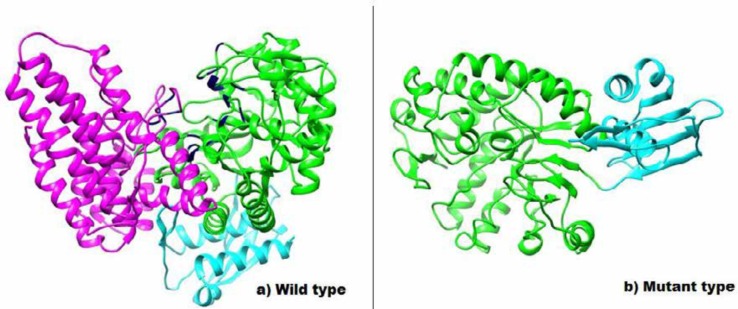
3D structure of the Alpha-N-acetylglucosaminidase and its truncated form. N-terminal domain: Blue, central domain: Green and C-terminal domain: Purple. In the truncated protein, the important residues in the active site of Alpha-N-acetylglucosaminidase located are in the cleft between central and C-terminal were missed. The glycosylation sites in N435, 503, 526 and 532 were also lost

**Figure 3:**
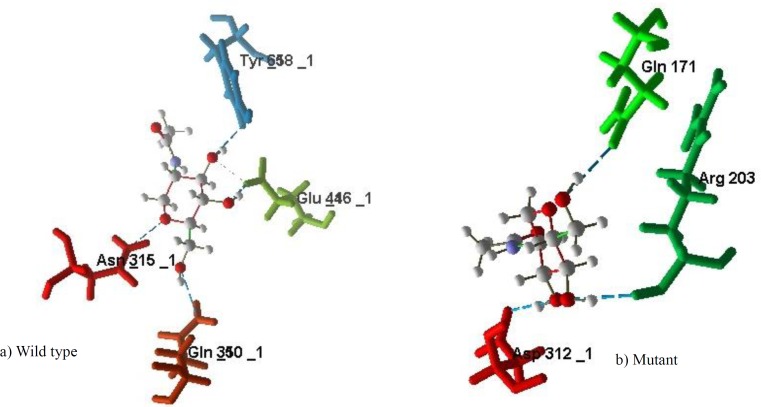
Diagram displaying the graphical representation of docking study of both types of the protein with N-Acetyl-D-Glucosamine ligand molecule. a) Docking study of the wild type of NAGLU protein. b) Docking study of the truncated form of NAGLU protein. All graphical representation was created through Molegro Virtual Docker (MVD).

The mutation was confirmed by Sanger sequencing and both parents were heterozygous for the detected variant. Segregation analysis in the pedigree was consistent with autosomal recessive nature of the disease and revealed that the variant was co-segregating with the disease (Figure 1). In silico protein analysis for detected variant indicated the loss of catalytic residue E446 in central domain (Domain II) and also all the residues in C terminal domain (Domain III) (Figure 2). In the truncated protein, the important residues R510, H512, R519, W649, I655 and Y658 in the active site of N-acetyl glucose amine located in the cleft between Domain II and III were missed. The glycosylation sites in N435, 503, 526 and 532 were also lost. Further analysis was made to determine how the novel frameshift variant could affect the enzyme function through binding to its ligands. Details of the docking study of intact protein and its truncated form with selected N-Acetyl-D-Glucosamine ligand molecule are shown in Figure 3. The Tyr 658, Glu 446, Asp 315 and Gln 350 from wild type protein as well as residues Gln 171, Arg 203 and Asp 312 from truncated protein are prominently involved in hydrogen bond interactions with the ligand. The binding affinity of the ligand toward target was evaluated based on their MolDock score. The comparative analysis of results obtained from both types of proteins is shown in Table 3.

 The MolDock scores of the truncated and the normal NAGLU proteins were significantly different especially for binding to N-Acetyl-D-Glucosamine ligand. The binding affinity of the truncated protein with N-Acetyl-D-Glucosamine was higher than the wild type protein, leading to disrupted interactions as well as reduced catalytic activities of the. However, the binding affinities of the other ligands against the two forms of the protein were not very different. 

**Table 1:** Used muccopolysaccharidose gene panel

Gene	RefSeq	Nucleic Acid Alteration	Amino Acid Alteration	Mutation Locatation	Zygocity	Chr-location
*NAGLU*	NM_000263	c.1294-1304 delCTCTTCCCCAA	p.432LeufsX25	CDS6	Het	Chr17:40695318.40695328

**Table 2:** Protein prediction analyses of the novel detected variant on *NAGLU* gene

Protein prediction algorithm	c. 1294-1304 delCTCTTCCCCAA, P. F 433 H fs*24
MutationTaster	Disease-causing/probably deleterious Truncated protein (might cause NMD) Splice site changes Frameshift Protein features (might be) affected
SIFT Indel	Damaging Frameshift
DDIG Indel	Disease causing

**Table 3:** The docking prediction results for the mutation in NAGLU gene using Molegro Virtual Docker software

	** Wild type**		**Mutant type**	
	**MolDock Score (kcal/mol)**	**Rerank score** **(kcal/mol)**	**MolDock Score (kcal/mol)**	Rerank score (kcal/mol)
**Beta-D-Mannose**	-66.9973	-67.44	-64.88142	-72.779
**D-Xylitol**	-66.14256	-59.481	-67.04284	-66.298
**Glycerol**	-45.81762	-43.207	-46.3082	-43.764
**N-Acetyl-D-Glucosamine**	-83.1905	-73.157	-72.23936	-23.896

## Discussion

Current study applied targeted NGS to Sanfilippo syndrome and reports a new frameshift variant (c.1294-1304 del CTCTTCCCCAA, p.432LeufsX25) in the *NAGLU* gene in a consanguineous family which subsequently validated by Sanger sequencing and in silico analysis of the putative variant. *NAGLU* gene code for a lysosomal enzyme called Alpha-N-acetylglucosaminidase (1) This gene encoded for as N-acetyl glucose amine, have been previously reported to cause mucopolysaccharidosis IIIB (MPS IIIB) (19) found at the cell surface and extracellular matrix binds to a variety of protein ligands and regulates a wide range of biological roles in key developmental signaling pathways. 

N-acetyl glucose amine is involved on degradation of heparan sulfate which is complex polysaccharide belonging to the glycosaminoglycan (GAG) family (20). Therefore, these molecules accumulate and impair normal cellular function (2). *NAGLU* gene is located on chromosome 17 (17q21.2) contains six exons which span approximately 8.3 kb and encodes a 743 amino acid protein (1, 21). More than 150 mutations have been reported in the *NAGLU* gene to date, the greater number of them are missense but also nonsense and Indels have been reported (HGMD, http: www.hgmd.org). In our study, 11 bp deletion variant, (c.1294-1304 del CTCTTCCCCAA, p.432LeufsX25), located on the sixth exon, caused a frameshift and decreased protein length from 743 amino acids to 454. The enzyme is composed of three domains. N-terminal domain of Alpha-N-acetylglucosaminidase containing the signal sequence (1aa-112aa), central domain, which has a time barrel fold (126 aa 456 aa) and C-terminal domain that has an alpha-helical fold (466aa 725aa). Exon six, the largest exon of *NAGLU*, encode for central tim Barrel and C-terminal domain consisting of 403 aa (341 to 743). Functional protein analysis for this variant indicated the loss of catalytic site residue in central domain and Cterminal domain (Figure 2). In the truncated protein, the important residues in the active site of Alpha-N-acetylglucosaminidase located is in the cleft between central and Cterminal were missed. The glycosylation sites in N435, 503, 526 and 532 were also lost. NAGLU protein involved is hydrolysis of terminal non-reducing N-acetyl-D-glucosamine residues in N-acetyl-alpha-D-glucosaminides. In docking study, binding affinity of the truncated protein with N-Acetyl-D-Glucosamine was higher than the wild type protein, leading to disrupted interactions as well as reduced catalytic activities of the NAGLU protein. In addition, the interruption in the interaction with other cooperating protein was responsible for the etiology.

Studies intended to elucidate genotype-phenotype correlation patterns have crucial importance in clinical predictive capabilities. However, few such correlations have been established due to high genetic and clinical heterogeneity in MPS IIIB affected alike with other forms of MPSs (4, 21-23). In our report, the girl’s signs and symptoms were severe, and she manifested most of Sanfilippo syndrome symptoms due to frameshift variant, which is compatible with other reports of severe form (4, 6, 21, 22, 24, 25). A minority of severe MPS IIIB affected has been reported and in most of them, the patient harbored nonsense mutations, insertions or deletion resulting in truncated protein (HGMD, http: www.hgmd.org). Patients with milder form of MPS IIIB are more common which it is in contrast with general notion severe forms most frequent (26). In this study, the novel variant leads to partial disruption of the protein sequence in central domain and complete loss of the cytoplasmic domain, producing a truncating protein and predicted cause loss-of-function through NMD according to some computational predictions. Moreover, docking analysis revealed different binding affinity for its ligand N-Acetyl-D-Glucosamine and therefore impaired hydrolysis function in truncated protein. Following evidence confirmed this variant pathogenicity in the base of ACMG guideline: null frameshift variant (PVS1), variant absence in control population (PM2), co-segregation with the disease (PP1), in silico analysis (PP3), coinciding clinical manifestation (PP4). According to the ACMG guideline, the reports of truncating variants downstream of the extreme 3 prime of a newly identified truncating variant are confirmatory for its pathogenicity. In NAGLU gene, downstream of (c.1294-1304 del CTCTTCCCCAA, p.432LeufsX25) variant, we have truncating variants related to the most severe MPS phenotype (rs745831568, rs771031938, rs886039895, rs749338526, rs778021009, rs35145991). 

Given the limitations of clinical tools and the variable phenotypic characteristics of MPS, using NGS and exome sequencing will facilitate quick and correct diagnosis and accelerate possible medical interventions. In addition, NGS notably reduce time and cost and assist to better identification of genotype-phenotype correlation and help to future approaches to move from bench to bed. Results can be worthwhile for genetic counseling in the family, cascade carrier screening and prevention in the next pregnancies by performing preimplantation genetic diagnosis

**In conclusion,** our study expands the spectrum of *NAGLU* pathogenic variants and confirms the utility of targeted NGS sequencing in genetic diagnosis and also the utility and power of additional family information.

## Author`s contribution

Mehdi Khorrami: Substantial contributions to the conception or design of the work; or the acquisition, analysis, or interpretation of data for the work.

Majid Kheirollahi: Drafting the work or revising it critically for important intellectual content, final approval of the version to be published 

Manijeh Mahdavi: Agreement to be accountable for all aspects of the work in ensuring that questions related to the accuracy or integrity of any part of the work are appropriately investigated and resolved

Fatemeh Fakhr: Drafting the work or revising it critically for important intellectual content, analysis, or interpretation of data for the work.

## References

[1] Zhao HG, Li HH, Bach G, Schmidtchen A, Neufeld EF (1996). The molecular basis of Sanfilippo syndrome type B. Proceedings of the National Academy of Sciences.

[2] Hara A, Kitazawa N, Taketomi T (1984). Abnormalities of glycosphingolipids in mucopolysaccharidosis type III B. Journal of lipid research.

[3] Weber B, Guo X-H, Kleijer WJ, van de Kamp JJ, Poorthuis BJ, Hopwood JJ (1999). Sanfilippo type B syndrome (mucopolysaccharidosis III B): allelic heterogeneity corresponds to the wide spectrum of clinical phenotypes. European Journal of Human Genetics.

[4] Bunge S, Knigge A, Steglich C, Kleijer WJ, van Diggelen OP, Beck M (1999). Mucopolysaccharidosis type IIIB (Sanfilippo B): identification of 18 novel α-N-acetylglucosaminidase gene mutations. Journal of medical genetics.

[5] Muenzer J (2004). The mucopolysaccharidoses: a heterogeneous group of disorders with variable pediatric presentations. The Journal of pediatrics.

[6] Valstar M, Ruijter G, Van Diggelen O, Poorthuis B, Wijburg F (2008). Sanfilippo syndrome: a mini-review. Journal of inherited metabolic disease.

[7] Cleary M, Wraith J (1993). Management of mucopolysaccharidosis type III. Archives of disease in childhood.

[8] Li H, Durbin R (2009). Fast and accurate short read alignment with Burrows–Wheeler transform. Bioinformatics.

[9] Schwarz JM, Rödelsperger C, Schuelke M, Seelow D (2010). MutationTaster evaluates disease-causing potential of sequence alterations. Nature methods.

[10] Hu J, Ng PC (2013). SIFT Indel: predictions for the functional effects of amino acid insertions/deletions in proteins. PloS one.

[11] Folkman L, Yang Y, Li Z, Stantic B, Sattar A, Mort M (2015). DDIG-in: detecting disease-causing genetic variations due to frameshifting indels and nonsense mutations employing sequence and structural properties at nucleotide and protein levels. Bioinformatics.

[12] Thomsen R, Christensen MH (2006). MolDock: a new technique for high-accuracy molecular docking. Journal of medicinal chemistry.

[13] Roy A, Kucukural A, Zhang Y (2010). I-TASSER: a unified platform for automated protein structure and function prediction. Nature protocols.

[14] Biasini M, Bienert S, Waterhouse A, Arnold K, Studer G, Schmidt T (2014). SWISS-MODEL: modelling protein tertiary and quaternary structure using evolutionary information. Nucleic acids research.

[15] Kiefer F, Arnold K, Künzli M, Bordoli L, Schwede T (2008). The SWISS-MODEL Repository and associated resources. Nucleic acids research.

[16] Arnold K, Bordoli L, Kopp J, Schwede T (2006). The SWISS-MODEL workspace: a web-based environment for protein structure homology modelling. Bioinformatics.

[17] Guex N, Peitsch MC, Schwede T (2009). Automated comparative protein structure modeling with SWISS‐MODEL and Swiss‐PdbViewer: A historical perspective. Electrophoresis.

[18] Richards S, Aziz N, Bale S, Bick D, Das S, Gastier-Foster J (2015). Standards and guidelines for the interpretation of sequence variants: a joint consensus recommendation of the American College of Medical Genetics and Genomics and the Association for Molecular Pathology. Genetics in medicine: official journal of the American College of Medical Genetics.

[19] O'brien JS (1972). Sanfilippo syndrome: profound deficiency of alpha-acetylglucosaminidase activity in organs and skin fibroblasts from type-B patients. Proceedings of the National Academy of Sciences.

[20] Varki A, Kornfeld S (1980). Identification of a rat liver alpha-N-acetylglucosaminyl phosphodiesterase capable of removing" blocking" alpha-N-acetylglucosamine residues from phosphorylated high mannose oligosaccharides of lysosomal enzymes. Journal of Biological Chemistry.

[21] Yogalingam G, Hopwood JJ (2001). Molecular genetics of mucopolysaccharidosis type IIIA and IIIB: Diagnostic, clinical, and biological implications. Human mutation.

[22] Gilkes J, Patterson B, Heldermon C (2014). Mucopolysaccharidosis III Molecular genetics and genotype-phenotype correlations. OA Genet..

[23] Kamp JVD, Niermeijer M, Figura K, Giesberts M (1981). Genetic heterogeneity and clinical variability in the Sanfilippo syndrome (types A, B, and C). Clinical genetics.

[24] Andria G, Natale P, Giudice E, Strisciuglio P, Murino P (1979). Sanfilippo B syndrome (MPS III B): mild and severe forms within the same sibship. Clinical genetics.

[25] Beesley C, Jackson M, Young E, Vellodi A, Winchester B (2005). Molecular defects in Sanfilippo syndrome type B (mucopolysaccharidosis IIIB). Journal of inherited metabolic disease.

[26] Valstar MJ, Bruggenwirth HT, Olmer R, Wevers RA, Verheijen FW, Poorthuis BJ (2010). Mucopolysaccharidosis type IIIB may predominantly present with an attenuated clinical phenotype. Journal of inherited metabolic disease.

